# Muller’s nobel prize research and peer review

**DOI:** 10.1186/s13010-018-0066-z

**Published:** 2018-10-19

**Authors:** Edward J Calabrese

**Affiliations:** Department of Environmental Health Sciences, Morrill I, N344, University of Massachusetts, Amherst, MA 01003 USA

**Keywords:** Gene mutation, X-ray, Nobel Prize, Hermann J. Muller, History of science, Peer review, Drosophila

## Abstract

**Background:**

This paper assesses possible reasons why Hermann J. Muller avoided peer-review of data that became the basis of his Nobel Prize award for producing gene mutations in male *Drosophila* by X-rays.

**Methods:**

Extensive correspondence between Muller and close associates and other materials were obtained from preserved papers to compliment extensive publications by and about Muller in the open literature. These were evaluated for potential historical insights that clarify why he avoided peer-review of his Nobel Prize findings.

**Results:**

This paper clarifies the basis of Muller’s (Muller HJ, Sci 66 84-87, 1927c) belief that he produced X-ray induced “gene” mutations in Drosophila. It then shows his belief was contemporaneously challenged by his longtime friend/confidant and Drosophila geneticist, Edgar Altenburg. Altenburg insisted that Muller may have simply poked large holes in chromosomes with massive doses of X-rays, and needed to provide proof of gene “point” mutations. Given the daunting and uncertain task to experimentally address this criticism, especially within the context of trying to become first to produce gene mutations, it is proposed that Muller purposely avoided peer-review while rushing to publish his paper in *Science* to claim discovery primacy without showing any data. The present paper also explores ethical issues surrounding these actions, including those of the editor of *Science*, James McKeen Catell and Altenburg, and their subsequent impact on the scientific and regulatory communities.

**Conclusion:**

This historical analysis suggests that Muller deliberately avoided peer-review on his most significant findings because he was extremely troubled by the insightful and serious criticism of Altenburg, which suggested he had not produced gene mutations as he claimed. Nonetheless, Muller manipulated this situation (i.e., publishing a discussion within *Science* with no data, publishing a poorly written non-peer reviewed conference proceedings with no methods and materials, and no references) due to both the widespread euphoria over his claim of gene mutation and confidence that Altenburg would not publically challenge him. This situation permitted Muller to achieve his goal to be the first to produce gene mutations while buying him time to later try to experimentally address Altenburg’s criticisms, and a possible way to avoid discovery of his questionable actions.

## Background

Hermann J. Muller [20] claimed that high doses of X-rays induced gene mutations in Drosophila in his paper entitled the “Artificial Transmutation of the Gene”, introducing the concept of point mutation (page 87, left column). In a recent paper, I argued that Muller deliberately avoided peer review of his Nobel Prize data to win the race to become the first to report such uniquely important findings [12]. However, further examination of the underlying reasons for Muller’s avoidance of the peer review process suggests it may not have been simply to win the race to publication. This paper offers a more nuanced and novel hypothesis that Muller feared his “landmark” paper may fail the peer review process since the critical gene mutation interpretation was not supported by data. This concern resulted in Muller devising a camouflaged process to avoid peer review while still retaining his worldwide acclaim thereby securing “primacy” for a discovery that would later yield a Nobel Prize.

## The new interpretation

Despite the rapid and massive acclaim Muller received following his presentation revealing X-ray induced gene mutation at the Fifth International Genetics Congress in September, 1927 [13], his striking gene mutation interpretation was not immune to criticism. In fact, Muller was acutely sensitive to insightful, yet private criticism, by his closest friend Edgar Altenburg [22] that he had “only” produced hundreds of transgenerational phenotypic changes with no proof of his gene mutation interpretation. While the production of massive numbers of such genetic changes in such a short period of time was overwhelming to many, for Altenburg it remained a potentially important observation in search of an explanation. He told Muller that he needed to show that the high dose mutation-inducing X-rays were not merely blasting holes in large sections of chromosomes, producing the heritable phenotypic changes via a chromosomal deletion rather than a gene mutation mediated process. For Altenburg this was the crucial question and Muller knew that he was correct.

For Muller this criticism was devastating as he had come so close to the goal of gene mutation discovery, only to be “denied” the satisfaction by his best friend, who offered a more objective perspective. While Muller needed to hear this criticism, he also was blinded by ambition to be first on perhaps the most critical question of the day in biology, that is, discovery of the mechanism of evolution.

In the years leading up to the key research of Muller on X-ray induced mutations he had made it clear that finding a means to induce mutation at the level of gene would be a profound advance as it may provide a mechanism that could drive evolution [8, 14]. Muller was dismissive of multiple claims of induced mutation during the early 1920s that were mediated at the level of chromosome rather than gene. Muller was firm that unless the induced changes occurred at the level of gene their significance would be very limited, not a qualitative advance. Altenburg was forcing Muller to be as self-critical as he was critical of numerous previously failed attempts by others to address the same issue.

Muller’s [19] ground breaking report on July 22, 1927 in the journal *Science* that he had induced hundreds of gene mutations by X-rays was based upon the production of allomorphic (i.e. visual) transgenerational phenotypic changes and lethality. He made this gene mutation claim/interpretation in an article that discussed his findings, but failed to include any data. The scientific community would have to wait until the second week of September that year at the International Genetics Congress for Muller to present the actual findings [20].

A review of his Nobel Prize data notebook (Muller, Lilly Library, University of Indiana-Bloomington) reveals that findings were derived from three experiments with the second experiment being wrapped up with respect to data collection in the laboratory at the end of May, 1927. The dates of the third experiment were not recorded in the lab notebook although Muller [22] indicated that he ran out of time to include a control group, probably in order to prepare for and travel to the Genetics Congress. The data discussed in *Science* matches most closely to that reported in experiment # 1 that was completed in the laboratory in December, 1926.

While it is unknown when Altenburg precisely offered his criticism to Muller, the actions of Muller to introduce a profound advance on one of the most significant questions confronting biology in the journal *Science* without any proof was very brash under any circumstances. Permitting this to occur was also similarly flamboyant for the editor, James McKeen Cattell. A careful evaluation of the entire preserved correspondence record of Cattell, as an individual and *Science* editor did not reveal the arrangement between him and Muller. Likewise, this is the case following a careful review of the Muller correspondence files.

That Muller had the personality to manipulate the scientific community and the general public can be seen in this Nobel Prize Lecture (December 12, 1946) where he deliberately deceived the audience arguing that there was no possibility of a threshold dose response for radiation induced mutations when he had just observed data supporting such a conclusion from Caspari and Stern from the University of Rochester, one month prior to the Nobel Prize Lecture [4, 5]. Muller offered this deception to promote his long held ideology in support of a linear non-threshold dose response for mutation and cancer risks. Muller [24, 25] would also deliberately offer inaccurate and deceptive comments in the scientific literature to promote linear non threshold (LNT), expressly contradicting private correspondence between himself and Stern [6]. Such high stakes risk taking and bluffing behavior of Muller surfaced two decades prior to his Nobel Prize Lecture, when he published a description of these findings without data, knowing that he lacked proof for his key gene mutation conclusion. Muller had the chance to submit this most significant research for peer review but choose not to do so. As pointed out, the conference proceedings paper with the data lacked a methods section, provided no references cited and even failed to acknowledge the prior recent paper (January, 1927) by Gager and Blakeslee [15] which provided what appears to be the first evidence of radiation induced gene mutation. Until this point in his career, Muller published his papers within journals with a well-established peer review record as he was guided into this scientific acculturation by his Columbia University mentor, Thomas Hunt Morgan. Yet, for the paper of greatest significance he bizarrely published a discussion of preliminary findings with no data and then avoided peer review with a poorly developed paper in the Congress proceedings [12].

It is likely that Muller was both devastated by the criticism of Altenburg and could not find any way to address it, at least not in the short term. Yet, he knew he was in a race to be first, even if Gager and Bakeslee may have some claim to the honor he could handle them with a series of Muller-like blistering challenges. It appears that Muller rolled the proverbial dice, ignored, at least for the time being, the criticisms of Altenburg, and went on to claim his “unequivocal” belief in having produced “gene” mutations, all the while knowing that he had not proven it. This was similar to his deceptive performance at the Nobel Prize Lecture, showing the same risk taking behavioral trait. Muller had confidence that his duplicity would not be revealed by those that knew of his scamming behaviors, such as Stern and Caspari and perhaps a few others at the University of Rochester for the Nobel Prize Lecture, and Altenburg for his Nobel Prize research.

Muller would find a means to address the criticism of Altenburg but it would take several years. He adopted the concept of reverse mutation and thought that it could provide indirect, but functional proof that his treatments would yield genes that would still be working and therefore not destroyed by the high doses of radiation via massive deletions. However, it is curious to note that Muller mentioned that he had not observed any reverse mutations in the *Science* paper. Thus, in his own experimental model and methods of mutation induction he had not observed the reverse mutation phenomenon. However, in the fall of 1927 he claimed to have observed two examples [18, 23] and then in early 1928 his colleague at Texas James Patterson found evidence of a third example [22]. These very limited observations offered Muller a glimmer of hope to perhaps address the criticism of Altenburg, but only time and experimental evidence would tell. Thus, even a year after the publication in *Science* Muller had no proof that he had induced gene mutation and was now going to try to support this interpretation via a collaboration with Patterson. At the December, 1927 [18] American Association for the Advancement of Science (AAAS) Conference in Nashville Muller provided the a glimpse into the emerging importance of the reverse mutation concept as he stated that the newly observed two reverse mutations should provide more information on gene structure than “gene” loses and be more significant in the study of evolution, the central theme of biology. In fact, the argument to support the claim of gene mutation would morph into that of reverse mutation [28].

This discussion provides context and a plausible explanation for why Muller deliberately avoided peer review. A case can be made that he accepted the validity of Altenburg’s concerns. This would have suggested to him that there was the possibility/probability of this paper being rejected. Muller was caught in an ethical dilemma: he could address the criticisms of Altenburg or finesse his manuscripts and presentations to avoid possible devastating critical reviews while accepting the uncritical acclaim of the scientific and world communities who were overwhelmed and transformed with his capacity to produce transgenerational phenotypic changes; this same community then accepted his gene mutation interpretation based largely on an appeal to authority.

In a September 1928 publication (based on an April 15, 1928 presentation at the National Academy of Sciences) in the Proceedings of the National Academy of Sciences (PNAS) Muller acknowledged the criticisms of Altenburg [22]. He also indicated that Patterson had found an example of a reverse mutation. Muller then stated that he was now going to follow up on the suggestion of his wife, a mathematician, to address the Altenburg criticism via the study of reverse mutation. At this point Muller was seemingly aware of only three cases of what might be X-ray induced reverse mutation in the PNAS paper. It is also curious that Muller never acknowledged the two examples of reverse mutation that he referenced in the AAAS presentation nine months earlier.

Nearly three years after the Altenburg criticism Patterson and Muller [28] would provide support for the gene mutation interpretation using the reverse gene mutation. On page 574 they concluded that “the above (reverse mutations) demonstrates that mutations can be produced in both of two opposite directions at the same locus.” They further claimed that these findings were “irreconcilable with the view that all mutational changes by X-rays consist of losses.”

In retrospect the original “proof” of gene mutation using alleomorphic phenotypic changes-as both an observation and explanation-was not convincing to Muller following the Altenburg criticism. Thus, overtime Muller came to use the reverse mutation observations as the means to explain the induced transgenerational phenotypic changes. In some ways, this was a type of scientific bait and switch process that was nearly unrecognizable, yet critically important over the issue of gene mutation discovery. Nonetheless, the scientific community was mesmerized by the findings and publicity of Muller to such an extent that it quickly and uncritically came to believe that Muller had produced gene mutations based on an interpretation lacking scientific justification.

While Muller may have thought that the reverse mutation findings may have salvaged the gene mutation interpretation, even though three years too late, it too would be challenged very seriously by another thoughtful equal peer of Muller, the corn geneticist Lewis Stadler at the University of Missouri. In fact, Stadler [31] had been only a few months behind Muller in the reporting of X-ray induced mutations. While Stadler at first accepted the gene mutation interpretation of Muller this would change some four years later.

In 1929 Barbara McClintock [16] developed a vastly improved cytogenetic method for assessing the characteristics and appearance of chromosomes of corn. At that time this technique was far more advanced than had been developed for Drosophila [8]. In 1931 [17], McClintock provided a cytogenetic evaluation of X-ray induced mutations in corn under the direction of Stadler. He had observed transgenerational phenotypic changes similar to those produced by Muller with Drosophila. However, McClintock’s advanced cytogenetic methods revealed that the so called “gene” mutations that Stadler believed he had produced were most likely gross chromosomal deletions. Thus, McClintock concluded that the doses of X-rays used by Stadler were simply punching holes in the chromosomes and that he had not induced gene mutations as he had thought. After some deliberation, Stadler [32, 33] came to accept the new insights of McClintock and soon came to question whether this was also the case with Muller’s gene mutations as first suggested by Altenburg. While Muller claimed that the X-ray induced reverse mutation explanation addressed the question, this position was not convincing to Stadler [34] and others (see [8] for a review) who offered a series of experimentally based alternative explanations (e.g. position effect, transpositional elemental effects) for Muller’s reverse mutation phenotype findings without the need for gene mutation).

Overlooked in this debate was the dissertation of Clarence P. Oliver, Muller’s student at the University of Texas. In his dissertation (i.e., Conclusion #10) [26] and in his journal publication (i.e., Conclusion #11) [27], Oliver stated “It is not possible to produce gene mutations without producing gene rearrangement, or vice versa”. This statement undercuts Muller’s conclusion that he produced “point mutations” at doses far higher than those used by Oliver [26]. Given this contradictory statement, it is curious that Muller approved this dissertation since this statement was very Stadler-like.

While most in the scientific community seemed to believe that Muller had induced gene mutation, Muller and Stadler would contest this point until Stadler died in 1954 with neither side conclusively winning this debate although the preponderance of the evidence favored Stadler [34]. In the end, Stadler adopted the perspective of Altenburg that Muller mistook an observation (i.e. transgenerational phenotypic changes) for an explanation (i.e., gene mutation). However, once nucleotide analysis methods were developed, he was shown to have achieved his transgenerational phenotypic changes via the “punching of holes” in chromosomes via modest to extraordinarily large deletions rather than via point mutations [8–11].

## Discussion

The actions of Muller and their effects on other key figures are worth exploring for their historical and ethical considerations. In the case of Muller, we find the tactic of publishing on a transformative topic such as gene mutation without providing data as self-serving and not abiding by the generally accepted culture of scientific responsibility of successfully passing a responsible peer review. This failure was also coupled with his acceptance of worldwide acclaim and hoped for professional job offers (see letter to Carl Hartman, October 28, 1927 [21]). Thus, Muller was masterful in manipulating the scientific community without his manipulation being exposed.

In the case of Cattell, he was a close friend of TH Morgan, both being long time professors at Columbia University [1]. It is likely that Muller used their past Columbia association to approach Cattell. At that time Cattell personally owned the journal *Science* and may have sought to profit from the likely intense positive publicity. Thus, in some ways Muller and Cattell exploited each other for personal profit at the expense of society. Approximately two decades later Cattell would transfer ownership of *Science* to the AAAS [30].

In the case of Altenburg, he appears as a loyal friend and would remain so until both he and Muller died in the same year (1967), a few months apart. Their relationship was unique as seen when Muller attempted suicide in 1932. His suicide note was directed to Altenburg rather than his family [14]. Thus, for Altenburg loyalty to a close friend may have overwhelmed his responsibility to science and the broader community. It is both interesting and ironic that Altenburg [2] would eventually publicly side with the Stadler-McClinctock perspective that the high doses of radiation were mediating Muller’s mutations most likely via McClintock’s inducible transpositional element (i.e. the basis of her own Nobel Prize in 1981). Furthermore, Ratner et al. [29] confirmed this perspective later in studies closely replicating Muller’s experiments.

## Conclusion

The Muller story is a troubling one of excessive and unbridled ambition, self-serving manipulation, high risk behavior and an enabling authority (i.e. *Science* editor Cattell) and a very loyal/protective friend (i.e. Altenburg) along with a scientific community that failed to demand accountability and a Nobel Prize committee that inadequately evaluated Muller’s findings. The implications of such actions are profound since it has been Muller’s incorrect gene mutation interpretation and its legacy that created the LNT dose response model, leading to its recommendation by the US National Academy of Sciences in 1956 [3] and then subsequently adopted by all regulatory programs throughout the world. Thus, Muller’s many significant deceptions facilitated a long, continuing and influential reach with a profound impact on society which continues to the present without any action, acknowledgment or change by the scientific community and government regulatory agencies.

The recent detailed historical assessment [6–8] and the last two vignettes (this paper and [12]), document significant new scientific and ethical insights into the career of Hermann Muller and this era. The set of papers reveal Muller to be an inspiring and principled leader while, at other times, he appears as a person with distorted self-serving interests, highly ideological and partisan, and with little evidence of a moral compass, all the while being very self-righteous, within the context of a very intelligent, unrelenting, aggressive and at times disagreeable manner. This combination can be a dangerous personal and societal cocktail, making him capable of achieving much and often at the expense of many. Despite the documented limitations and flaws of Muller, the most significant criticisms and concern should be directed to the scientific and regulatory communities, such as the US Environmental Protection Agency, that have uncritically adopted and sustained Muller’s findings as the foundation for cancer risk assessment and permitted this process to be corrupted by dominating ideological perspectives, failing in their congressionally mandated societal leadership.

## Abbreviations

AAAS: American Association for the Advancement of Science
LNT: Linear non Threshold
PNAS: Proceedings of the National Academy of Sciences
